# Association of Corneal Biomechanical Properties with Fingertip-Measured Advanced Glycation End Products and Carotenoids in Glaucoma Patients

**DOI:** 10.3390/jcm15020783

**Published:** 2026-01-18

**Authors:** Keigo Takagi, Hinako Ohtani, Chisako Ida, Mizuki Koike, Kana Murakami, Masaki Tanito

**Affiliations:** Department of Ophthalmology, Shimane University Faculty of Medicine, Izumo 693-8501, Japan; takagi918k@med.shimane-u.ac.jp (K.T.);

**Keywords:** advanced glycation end products, carotenoids, corneal biomechanical properties, ocular response analyzer, glaucoma

## Abstract

**Background/Objectives:** Advanced glycation end products (AGEs) and carotenoids are systemic indicators of metabolic and oxidative status, yet their influence on ocular tissue biomechanics remains unclear. This study investigated the relationships between systemic AGEs and skin carotenoid levels, as well as corneal biomechanical properties in glaucoma patients. **Methods:** A retrospective observational analysis was performed on 676 patients (1278 eyes) who attended the glaucoma clinic at Shimane University Hospital between May 2019 and August 2024. Fingertip skin autofluorescence (sAF)-based AGE scores using AGE Sensor^®^ and skin carotenoid scores using the Veggie Meter^®^ were collected as part of systemic evaluation. Corneal hysteresis (CH), corneal resistance factor (CRF), Goldmann-correlated intraocular pressure (IOPg), and corneal compensated intraocular pressure (IOPcc) were measured using the ocular response analyzer (ORA). Associations between systemic variables, AGEs, carotenoids, and ORA parameters were analyzed using univariate tests, mixed-effects regression models, and quartile-based comparisons. **Results:** The mean AGEs and carotenoid scores were 0.42 ± 0.10 arbitrary units and 338.5 ± 130.8 optical density units, respectively. Via a univariate analysis, an inverse association was found between carotenoid level and CRF; however, via multivariate analyses, neither AGEs nor carotenoid levels were associated with IOPg, IOPcc, CH, or CRF in any analysis. In contrast, demographic parameters showed significant associations with ORA parameters. Via quartile-based comparisons, a significant inverse correlation was found between AGEs and carotenoids (*p* < 0.0001). **Conclusions:** In conclusion, sAF-measured AGEs and skin carotenoids showed no remarkable associations with corneal biomechanical properties. AGEs and carotenoids demonstrated an inverse relationship with each other, and each marker was associated with several demographic parameters.

## 1. Introduction

Advanced glycation end products (AGEs) are formed when proteins, nucleic acids, lipids, and other macromolecules undergo nonenzymatic glycation (Maillard reaction) [1]. The accumulation of AGEs is associated with metabolic disorders such as diabetes [2] and physiological changes related to aging [3]. Additionally, AGEs may contribute to the progression of age-related chronic diseases, including cardiovascular disorders, heart failure, chronic kidney disease, and dementia [3,4,5]. In ocular diseases, AGEs are thought to play important roles in the pathogenesis of cataracts, diabetic retinopathy, age-related macular degeneration, and keratoconus [1,5,6,7]. In keratoconus specifically, increased expression of the receptor for AGEs has been reported in the corneal epithelium and tear film of patients compared with healthy controls [7]. Moreover, donor eyes from patients with glaucoma have shown greater AGE accumulation in the retina and optic nerve head compared with healthy controls [8]. In addition, age-related increases in total collagen content within the lamina cribrosa have been reported [9], indicating that aging-associated structural remodeling occurs in this region. Together, these findings suggest that AGE deposition and age-related collagen changes may reflect aging processes within the optic nerve that contribute to glaucomatous damage.

AGEs in bodily fluids and tissues can be measured using an enzyme-linked immunosorbent assay (ELISA), high-performance liquid chromatography (HPLC), or methods utilizing skin autofluorescence (sAF) [10]. sAF provides a simple and noninvasive means of assessing AGEs in clinical research settings, where direct biochemical sampling is often impractical. Notably, sAF values show positive correlations with both fluorescent and nonfluorescent AGE levels. [11,12]. Carotenoids such as lutein and zeaxanthin are antioxidant pigments concentrated in the fovea that reduce oxidative stress by scavenging free radicals and absorbing visible light [11,13,14]. Because humans cannot synthesize these pigments, dietary intake from vegetables and fruits is essential [15]. Epidemiological studies have shown a lower prevalence of glaucoma in populations with high fruit and vegetable consumption [16]. Recently, noninvasive fingertip reflection spectroscopy has enabled convenient monitoring of skin carotenoid levels [17]. Previous studies have reported a negative correlation between AGEs and carotenoids [11]. This finding suggests that carotenoid intake may help suppress oxidative and glycation processes, thereby reducing AGE accumulation [18].

Biomechanical properties refer to the stiffness and strength of biological tissues within their elastic range when subjected to loading [19]. The biomechanical properties of the cornea are represented by corneal hysteresis (CH) and corneal resistance factor (CRF) [19]. These parameters play important roles in refractive correction, the onset and progression of glaucoma [20,21,22], and the diagnosis and long-term monitoring of keratoconus [23]. Hysteresis represents the ability of biological tissues to dampen pressure changes caused by external forces [19]. The extracellular matrix components of the cornea, sclera, and lamina cribrosa are interrelated between the anterior and posterior segments of the eye; therefore, CH may also reflect the viscoelasticity of the sclera, peripapillary region, and lamina cribrosa [24]. CRF represents the stiffness and resistance of the cornea and has a significant positive correlation with central corneal thickness [25]. In glaucoma patients, both CH and CRF are significantly lower and may further decrease with disease progression [26], making them promising parameters for evaluating glaucoma beyond intraocular pressure. Corneal biomechanical properties can be measured using the ocular response analyzer (ORA, Reichert Technologies, Depew, NY, USA) and Corvis ST tonometry (Oculus Optikgeräte GmbH, Wetzlar, Germany) [27]. ORA evaluates corneal responses by measuring outward and inward applanation pressures during an air puff [19], whereas Corvis ST quantitatively measures corneal deformation using an ultra-high-speed Scheimpflug camera [27].

CH and CRF are indicators that reflect the structural and compositional properties of corneal collagen fibers. Previous studies have reported the accumulation of AGEs in the sclera and pinguecula of the human eye [28,29], as well as in the epithelial basement membrane of the cornea in patients with diabetes [30]. Because AGEs are involved in collagen glycation and cross-linking, they can induce tissue stiffening and loss of elasticity. Taken together, AGE accumulation may alter the biomechanical characteristics of corneal collagen tissue and, consequently, be associated with CH and CRF. Previous studies have shown age-related declines in CH [31], which may be attributed to progressive collagen glycation and AGE-induced cross-linking. In addition, skin carotenoid levels, which reflect systemic antioxidant status and correlate with collagen integrity, may counteract AGE-related tissue stiffening by suppressing oxidative reactions in the glycation pathway [32].

AGEs are known to induce collagen cross-linking and tissue stiffening, suggesting a potential influence on corneal stromal biomechanics, although the extent to which systemically measured AGEs reflect corneal tissue status remains unclear. Carotenoids, as systemic markers of antioxidant capacity, may be associated with tissue viscoelastic properties through their relationship with oxidative stress, but their relevance to corneal biomechanics has not been well explored. In this study, we examined the relationships between ORA parameters, systemic AGEs, and carotenoid scores in glaucoma patients to clarify whether systemic metabolic and oxidative statuses influence corneal biomechanical characteristics.

## 2. Materials and Methods

### 2.1. Subjects

**Figure 1:**
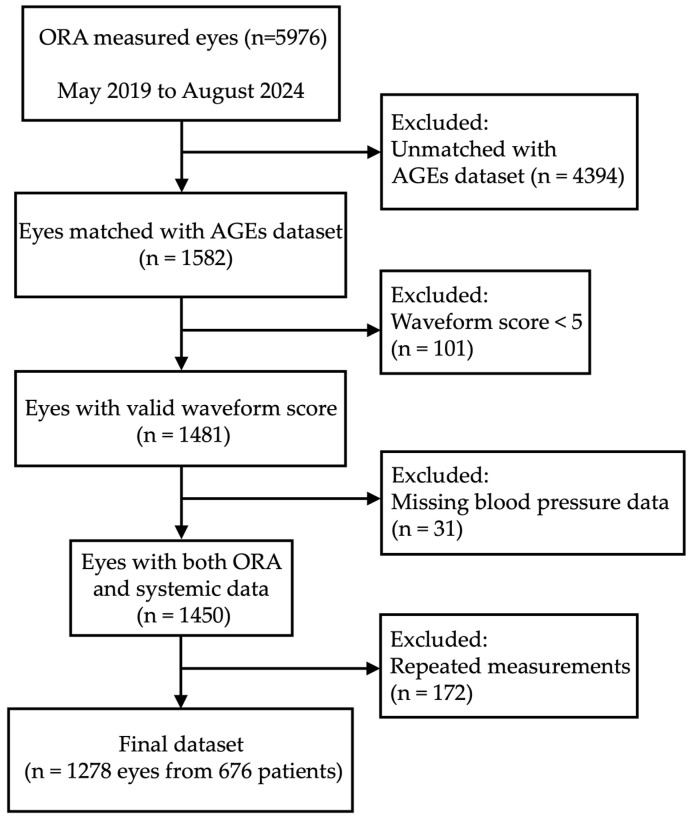
Flowchart of subject selection.

This retrospective observational case series study adhered to the tenets of the Declaration of Helsinki. The institutional review board (IRB) of Shimane University Faculty of Medicine reviewed and approved the research conducted at Shimane University Hospital (IRB No. 20200228-2; approval date of updated protocol: 17 October 2024). The IRB did not require individual written informed consent for publication; instead, the study protocol was posted at the study institution to notify participants about the study. At our institution, as part of the systemic evaluation for patients attending the glaucoma clinic, height, weight, blood pressure (BP), and pulse rate (PR) are measured, and both AGEs and carotenoid scores are routinely assessed at least once at the initial visit. BP was measured on the upper arm (HBP-9020, OMRON Healthcare Co., Ltd., Kyoto, Japan). Similarly, intraocular pressure and corneal biomechanical properties were evaluated using the ORA at least once at the initial visit. We included 676 patients who visited the ophthalmology department at Shimane University Hospital between May 2019 and August 2024, primarily from the glaucoma outpatient clinic. The data selection process was as follows: Of the 5976 eyes measured using the ORA during this period, 1582 eyes had matching patient IDs and measurement dates in the AGE dataset, while 4394 eyes did not. Among the subjects with both ORA and AGE measurements available, 101 cases with ORA waveform scores below 5 and 31 cases without blood pressure data were excluded. This resulted in 1450 eyes from 676 patients. Finally, to avoid duplication across multiple visits, only the first available measurement for each eye was used, and 172 eyes with repeated measurements were excluded. Thus, 1278 eyes from 676 patients were included in the final analysis (Figure 1). All measurements of AGEs, carotenoids, and ORA parameters were performed by one of thirteen experienced ophthalmic examiners.

### 2.2. Measurement of AGEs in the Fingertip Skin

The sAF was measured using an AGE sensor (Air Water Biodesign Inc., Kobe, Japan) and expressed as an AGE score. Measurements were taken on the middle finger of the non-dominant hand, which has the least amount of skin melanin [33]. During the measurement, the fingertip was mildly compressed at the distal part of the distal interphalangeal joint. This is a suitable area to avoid nonspecific sAF [30]. The measurements were obtained using excitation (365 nm) and emission (440 nm) wavelengths. The AGE score was expressed in arbitrary units (A.U.s). These measurements were performed two or three times, and the average score was used as the individual value. Our pilot study showed that the coefficient of variation and the intraclass correlation coefficient (Cronbach’s alpha) of the three repeated AGE measurements were 6.7 ± 7.3% and 0.938, respectively [34]. The measured AGEs were expressed as an AGE score in arbitrary units, ranging from 0.0 or 0.5 to an upper limit of 10.0. One arbitrary unit corresponds to the average AGE score of a healthy 50-year-old Japanese individual [35].

### 2.3. Measurement of Carotenoids in the Fingertip Skin

The carotenoid score was assessed with a Veggie Meter^®^ (Longevity Link Corporation, Salt Lake City, UT, USA), which uses pressure-mediated reflectance spectroscopy with a white-light-emitting diode spanning 350–850 nm [36]. Measurements were performed by experienced examiners, and results were reported as optical density (O.D.) units. These measurements were performed two or three times, and the average score was used as the individual value. The device was calibrated with manufacturer-provided reference materials before the start of each morning and afternoon session to ensure measurement stability. During acquisition, participants inserted the left middle finger into the instrument’s cradle to standardize tissue compression and optical coupling. The carotenoid score obtained by this method has been shown to correlate with circulating carotenoid concentrations and therefore provide a practical proxy for habitual vegetable and fruit intake [37]. The average carotenoid score of Japanese individuals aged 69.7 years was reported to be 343.1 ± 142.1 OD [17].

### 2.4. Measurement of ORA Parameters

**Figure 2:**
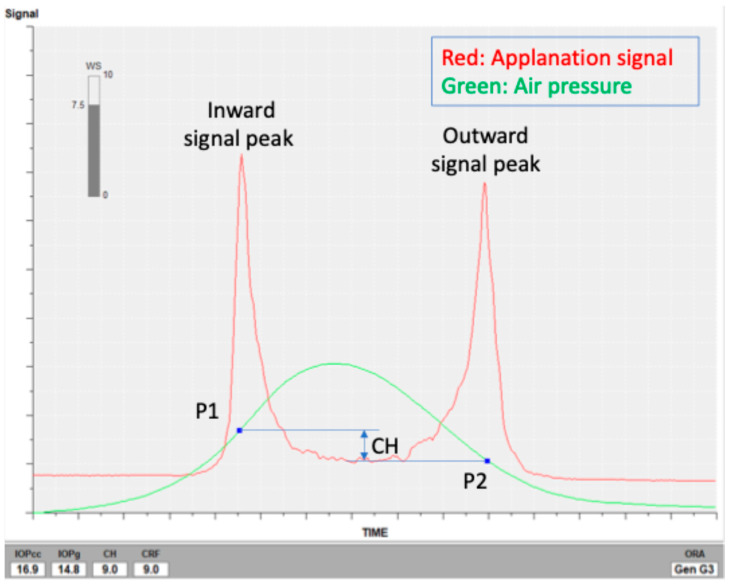
Representative ORA signal. IOPcc, corneal compensated IOP; IOPg, Goldmann-correlated IOP; CH, corneal hysteresis; CRF, corneal resistance factor; WS, waveform score; P1, first applanation pressure; P2, second applanation pressure.

ORA has the ability to measure Goldmann-correlated IOP (IOPg) and corneal compensated IOP (IOPcc) in addition to CH and CRF. The principles of ORA are based on non-contact tonometry, in which IOP is determined by the air pressure required to flatten the central cornea [38]. ORA calculates corneal biomechanical properties by quantifying the inward and outward responses of the cornea as it is deformed by air pulses over a time range of approximately 20 ms. The external force causes the cornea to press in slightly beyond the applanation pressure point and then return to its original shape. The inward and outward applanation pressure points are P1 and P2, respectively, and each parameter is calculated by using those points. IOPg is calculated as the mean of the first (P1) and second (P2) applanation pressures (Figure 2). IOPcc is corrected by CH and calculated as P2 − 0.43 × CH. CH represents the difference between these two pressure measurements (i.e., P1–P2). CRF is calculated using the formula P1 − kP2, where k is an empirically determined constant based on the relationship between both pressure measurements and central corneal thickness. This coefficient is usually calculated to be around 0.7 [39]. Each measurement was performed three times, and the dataset with the highest waveform quality index was selected for analysis.

### 2.5. Statistical Analysis

An analysis was conducted to examine the associations between systemic factors [age, sex, body mass index (BMI), sBP, dBP, and PR], AGE, carotenoid, and ORA parameters (IOPg, IOPcc, CH, and CRF). Univariate analysis was performed using Pearson’s correlation coefficient to assess relationships between continuous variables, while Student’s *t*-test was employed for categorical variables. To correct for bias introduced by including both eyes from the same patient, mixed-effects regression models were constructed to examine the relationships between each dependent variable and all independent variables in multivariate analysis. Specifically, separate mixed-effects models were built for each ORA parameter (IOPg, IOPcc, CH, and CRF) as a dependent variable. In each model, age (years), sex (M/F), BMI (kg/m^2^), sBP (mmHg), dBP (mmHg), PR (bpm), AGEs (A.U.), and carotenoids (O.D.) were included as fixed-effect covariates, while patient identity was treated as a random effect to account for within-subject correlation between eyes. These covariates were selected a priori based on their known or potential associations with corneal biomechanics and systemic metabolic or hemodynamic status. Additionally, the AGE and carotenoid scores were divided into quartiles (Q1–Q4), and the correlations between these quartile groups and independent variables were analyzed using the Cochran–Armitage trend test to assess linear trends across ordered groups. Statistical significance levels were set at the 5% and 1% levels, corresponding to p-values less than 0.05 and 0.01, respectively. All statistical analyses were performed using JMP Student Edition version 18 (SAS Institute Inc., Cary, NC, USA).

## 3. Results

Table 1 and Table 2 present the baseline characteristics of the subject-based and eye-based parameters, respectively. The mean ± SD of AGEs was 0.42 ± 0.1 A.U., and that of carotenoids was 338.2 ± 130.5 O.D. The mean ± SD values of the eye-based parameters were as follows: IOPg, 15.2 ± 6.3 mmHg; IOPcc, 17.1 ± 6.2 mmHg; CH, 9.2 ± 1.6 mmHg; and CRF, 9.3 ± 1.9 mmHg.

Table 3 shows the results of univariate analyses assessing the associations between demographic variables and ORA parameters. AGEs showed no significant correlations with any of the ORA parameters. Carotenoids exhibited a significant negative correlation with CRF (r = −0.001). Among the systemic factors, age showed significant negative correlations with IOPg, CH, and CRF (r = −0.03, −0.03, and −0.04, respectively). BMI showed significant positive correlations with IOPg, IOPcc, and CRF (r = 0.20, 0.17, and 0.06, respectively). sBP showed significant positive correlations with IOPg, IOPcc, and CRF (r = 0.04, 0.04, and 0.01, respectively) and a significant negative correlation with CH (r = −0.06). dBP showed significant positive correlations with IOPg, IOPcc, and CRF (r = 0.06, 0.06, and 0.02, respectively). PR showed significant positive correlations with IOPg, IOPcc, and CRF (r = 0.06, 0.05, and 0.02, respectively). Table 4 shows the results of univariate analyses assessing the associations between sex and ORA parameters. IOPcc and CH were significantly lower in males than in females (*p* = 0.015 and *p* < 0.0001, respectively).

Table 5 shows the results of multivariate analyses assessing associations between IOPg and AGEs, carotenoids, and other demographic parameters. No significant correlations were found with AGEs or carotenoids. IOPg showed a negative correlation with age (Est = −0.04, *p* = 0.012) and positive correlations with BMI, sBP, and PR (Est = 0.15, *p* = 0.012; Est = 0.04, *p* = 0.004; Est = 0.05, *p* = 0.003, respectively). Table 6 shows the results of multivariate analyses assessing associations between IOPcc and AGEs, carotenoids, and other demographic parameters. No significant correlations were found with AGEs or carotenoids. IOPcc showed positive correlations with BMI, sBP, and PR (Est = 0.13, *p* = 0.03; Est = 0.03, *p* = 0.015; Est = 0.04, *p* = 0.011, respectively). Table 7 shows the results of multivariate analyses assessing associations between CH and AGEs, carotenoids, and other demographic parameters. No significant correlations were found with AGEs or carotenoids. CH showed negative correlations with age and sex when being male was coded as positive (Est = −0.03, *p* < 0.0001; Est = −0.22, *p* = 0.0001, respectively). Table 8 shows the results of multivariate analyses assessing associations between CRF and AGEs, carotenoids, and other demographic parameters. No significant correlations were found with AGEs or carotenoids. CRF showed negative correlations with age and sex when being male was coded as positive (Est = −0.04, *p* < 0.0001; Est = −0.14, *p* = 0.04, respectively), and positive correlations with BMI, sBP, and PR (Est = 0.04, *p* = 0.02; Est = 0.01, *p* = 0.003; Est = 0.02, *p* = 0.003, respectively).

Table 9 shows the correlations of the AGE score, stratified by quartiles, with demographic parameters and ORA parameters. A significant inverse relationship was observed between AGE quartiles and carotenoid score (*p* < 0.0001). All parameters measured by ORA showed no significant correlation across AGE score quartiles. Significant associations were observed in the age, sex, and BMI across AGE score quartiles (*p* = 0.002, <0.0001, and <0.0001, respectively). Table 10 shows the correlations between carotenoid score, stratified by quartiles, with demographic parameters and ORA parameters. A significant inverse relationship was observed between carotenoid quartiles and AGE scores (*p* < 0.0001). All parameters measured by ORA showed no significant correlation across carotenoid score quartiles. Significant associations were observed in the age, sex, BMI, and dBP across carotenoid score quartiles (*p* = 0.0002, <0.0001, <0.0001, and 0.017, respectively).

## 4. Discussion

In this study, we found a significant inverse relationship between AGE score and carotenoid score. In multivariate analyses, neither AGE scores nor carotenoid scores showed significant associations with any of the ORA parameters. Interestingly, several demographic parameters demonstrated significant associations with specific ORA parameters in the multivariate analysis.

sAF-measured AGE scores showed no significant associations with ORA parameters, indicating that systemic AGEs may not have a direct effect on corneal biomechanical properties. ORA parameters such as CH and CRF reflect the structural characteristics of the cornea and peripapillary tissues and have been associated with glaucoma severity and progression [22]. Similarly, AGEs have been reported to accumulate in the optic nerve head of glaucomatous eyes, suggesting that systemic AGEs may contribute to ocular structural changes [8]. Based on this rationale, we examined whether systemic AGEs were related to corneal biomechanics in a large clinical cohort; however, no such associations were observed. One explanation is that sAF values may not adequately reflect tissue-specific AGE accumulation within the cornea. Previous histological studies using cadaveric diabetic corneas have demonstrated the presence of specific AGEs, including carboxymethyl-lysine, pentosidine, and imidazolone, within epithelial and stromal tissues [40,41]. Although direct corneal measurement using cadaver eyes might have yielded different results, it is not feasible in clinical studies involving living subjects. Despite these limitations, this study is the first to assess the relationship between sAF-derived AGEs and ORA parameters in a clinical population, and we believe it provides meaningful clinical value as an initial report in this field.

Carotenoids showed a significant negative correlation with CRF in univariate analysis, but this association disappeared in multivariate analysis. This suggests that carotenoids are unlikely to be directly related to CRF. As shown in Table 10, carotenoid quartiles were also associated with several demographic parameters, and these findings were consistent with previous studies [32,42,43]. This supports the usefulness of carotenoid measurements, as they reflect vegetable intake and systemic metabolic status. Based on these systemic correlations, the lack of an association with corneal biomechanical parameters suggests that dietary carotenoid intake does not influence corneal biomechanics. Carotenoids are natural pigments distributed widely in plants, including vegetables and fruits [44]. Because they are pigments, carotenoids are considered unlikely to accumulate in optically transparent tissues such as the cornea, supporting the biological plausibility of the absence of a direct association in this study. However, carotenoids may still have local effects within ocular tissues, which were not evaluated in this study because carotenoid distribution in the cornea has not been directly measured. Methods to assess tissue-specific carotenoid accumulation may be needed in future research. Carotenoids also showed a significant inverse correlation with AGEs, consistent with earlier reports [11]. This relationship suggests that dietary carotenoids may reduce AGE formation by suppressing oxidative reactions [45].

Several significant associations were identified between systemic demographic parameters and ORA parameters in the multivariate analysis. IOPg and IOPcc were positively associated with BMI, sBP, and PR, whereas IOPg was negatively associated with age. CH and CRF were lower in older and male participants, and CRF was positively associated with BMI, sBP, and PR. These findings are consistent with previous large-scale studies, including the UK Biobank, which demonstrated that blood pressure and heart rate increase pressure-based indices, whereas aging and male sex decrease corneal viscoelasticity [31,46]. This consistency supports the validity and representativeness of the dataset analyzed in the present study. Corneal biomechanics are known to be influenced by demographic factors, and AGEs are likewise affected by these variables. Therefore, evaluating AGEs in relation to CH, a tissue-specific parameter, is methodologically appropriate and constitutes a justified comparison. From a mechanistic perspective, age-related collagen remodeling, changes in tissue hydration associated with BMI and BP, and autonomic influences reflected by pulse rate may all modulate corneal biomechanical responses. Although these pathways were not the primary focus of the present study, such demographic effects on corneal viscoelasticity could partially influence glaucoma risk stratification through their impact on pressure- and biomechanics-based indices. Accordingly, these factors were treated as covariates rather than central explanatory variables in the current analysis.

This study has several limitations. First, it was retrospective in design and essentially cross-sectional; although multiple measurements were obtained from the same participants, longitudinal comparisons were not performed. Second, glaucoma subtype, disease severity, treatment history, and central corneal thickness were not evaluated. Although central corneal thickness and CH/CRF are modestly correlated, they represent distinct aspects of corneal biomechanics, with CH/CRF reflecting viscoelastic properties that cannot be fully explained by corneal thickness alone [25,47]. Third, participants were not stratified according to systemic comorbidities such as diabetes or hypertension. Nevertheless, this study included a very large sample size. As described above, consistent associations were detected between demographic characteristics and ORA parameters, and also with AGEs or carotenoids. We therefore believe that the overall integrity and internal validity of this dataset support the reliability of the present findings. Future studies could further investigate the relationships between AGEs, carotenoids, and corneal biomechanics in specific glaucoma subtypes such as exfoliation glaucoma or normal-tension glaucoma, as well as across different levels of glaucoma severity. In addition, potential discordance between measurement sites should be considered. Systemic AGEs assessed by sAF and skin carotenoid levels may not directly reflect their accumulation or biological effects within the avascular corneal stroma, and their influence on corneal biomechanics may be indirect or non-linear. It is also possible that threshold effects exist, whereby systemic metabolic or oxidative status affects corneal viscoelastic properties only beyond certain levels, which could not be evaluated in the present analysis.

## 5. Conclusions

This study is unique in that it investigates the relationship between corneal biomechanical properties and AGEs using a large clinical dataset. In both univariate and multivariable analyses, no significant correlations were found between corneal biomechanical parameters and AGEs. Further investigations using tissue-level AGE quantification and stratified analyses of disease subgroups are warranted to validate and refine these observations.

## Figures and Tables

**Table 1:** Demographic subject parameters (*n* = 676 subjects).

Parameters	Mean ± SD or *n* (%)	95% CI or *n* (%)
Age, years	68.7 ± 13.3	68.0, 69.4
Sex, male/female	373 (55)	303 (45)
BMI, kg/m^2^	23.0 ± 3.6	22.8, 23.2
sBP, mmHg	145.5 ± 22.1	144.3, 146.7
dBP, mmHg	81.8 ± 14.3	81.0, 82.5
PR, bpm	74.9 ± 12.6	74.3, 75.6
AGEs, A.U.	0.42 ± 0.1	0.42, 0.43
Carotenoids, O.D.	338.5 ± 130.8	331.3, 345.7

Data are expressed in mean ± SD for continuous variables, and in *n* (%) for qualitative variables. SD, standard deviation; BMI, body mass index; sBP, systolic blood pressure; dBP, diastolic blood pressure; PR, pulse rate; bpm, beats per minute; AGEs, advanced glycation end products; A.U., arbitrary unit; O.D., optical density.

**Table 2:** ORA parameters (*n* = 1278 eyes).

Parameters	Mean ± SD	95% CI
IOPg, mmHg	15.3 ± 6.3	14.9, 15.6
IOPcc, mmHg	17.1 ± 6.2	16.8, 17.5
CH, mmHg	9.2 ± 1.6	9.1, 9.3
CRF, mmHg	9.3 ± 1.9	9.2, 9.4

Data are expressed in mean ± SD. SD, standard deviation; IOP, intraocular pressure measured by Goldmann applanation tonometry; IOPcc, corneal compensated IOP; IOPg, Goldmann-correlated IOP; CH, corneal hysteresis; CRF, corneal resistance factor.

**Table 3:** Possible associations among items measured by ORA and various continuous parameters.

Parameters	IOPg			IOPcc			CH			CRF		
	r	95% CI	*p*-Value	r	95% CI	*p*-Value	r	95% CI	*p*-Value	r	95% CI	*p*-Value
Age, years	−0.03	−0.06, −0.01	0.012 *	0.01	−0.01, 0.03	0.62	−0.03	−0.04, −0.03	<0.001 **	−0.04	−0.05, −0.03	<0.001 **
BMI, kg/m^2^	0.20	0.11, 0.3	<0.0001 **	0.17	0.08, 0.27	0.0003 **	0.00	−0.03, 0.02	0.97	0.06	0.03, 0.09	<0.0001 **
sBP, mmHg	0.04	0.03, 0.06	<0.0001 **	0.04	0.03, 0.06	<0.0001 **	−0.06	−0.01, 0.00	0.008 **	0.01	0.00, 0.01	0.002 **
dBP, mmHg	0.06	0.04, 0.09	<0.0001 **	0.06	0.03, 0.08	<0.0001 **	0.00	−0.01, 0.00	0.30	0.02	0.01, 0.02	<0.0001 **
PR, bpm	0.06	0.04, 0.09	<0.0001 **	0.05	0.02, 0.08	0.0003 **	0.00	0.00, 0.01	0.25	0.02	0.01, 0.03	<0.0001 **
AGEs, A.U.	1.30	−3.5, 6.1	0.59	1.68	−3.1, 6.4	0.49	−0.52	−1.8, 0.74	0.42	−0.05	−1.5, 1.4	0.95
Carotenoids, O.D.	−0.001	−0.004, 0.001	0.27	−0.001	−0.003, 0.002	0.63	−0.001	−0.001, 0.00	0.09	−0.001	−0.002, 0.00	0.02 *

The correlation coefficient (r) is Pearson’s correlation coefficient. * and ** indicate significance levels at 5% (*p* < 0.05) and 1% (*p* < 0.01), respectively. ORA, ocular response analyzer; IOP, intraocular pressure measured by Goldmann applanation tonometry; IOPg, Goldmann-correlated IOP; IOPcc, corneal compensated IOP; CH, corneal hysteresis; CRF, corneal resistance factor; CI, confidence interval; BMI, body mass index; sBP, systolic blood pressure; dBP, diastolic blood pressure; PR, pulse rate; bpm, beats per minute; AGEs, advanced glycation end products; A.U., arbitrary unit; O.D., optical density.

**Table 4:** Possible associations among items measured by ORA and categorical parameters.

Parameters	IOPg			IOPcc			CH			CRF		
Sex	Male	Female	*p*-Value	Male	Female	*p*-Value	Male	Female	*p*-Value	Male	Female	*p*-Value
Mean ± SD	15.5 ± 0.2	15.0 ± 0.3	0.18	17.5 ± 0.2	16.7 ± 0.3	0.015 *	9.0 ± 0.1	9.4 ± 0.1	<0.0001 **	9.2 ± 0.1	9.4 ± 0.1	0.06
95% CI	15.0, 16.0	14.5, 15.5		17.0, 18.0	16.2, 17.2		8.9, 9.1	9.3, 9.5		9.1, 9.3	9.2, 9.6	

*p*-values were estimated by using Student’s *t*-test. * and ** indicate significance levels at 5% (*p* < 0.05) and 1% (*p* < 0.01), respectively. ORA, ocular response analyzer; IOP, intraocular pressure measured by Goldmann applanation tonometry; IOPg, Goldmann-correlated IOP; IOPcc, corneal compensated IOP; CH, corneal hysteresis; CRF, corneal resistance factor; SD, standard deviation; CI, confidence index.

**Table 5:** Multivariate analysis of possible associations between ORA-measured IOPg and demographic parameters.

Parameters	Estimate	Lower 95% CI	Upper 95% CI	*p*-Value
Age, years	−0.04	−0.07	−0.01	0.012 *
Sex, M/F	0.19	−0.23	0.60	0.38
BMI, kg/m^2^	0.15	0.03	0.26	0.012 *
sBP, mmHg	0.04	0.01	0.06	0.004 **
dBP, mmHg	0.01	−0.03	0.05	0.57
PR, bpm	0.05	0.02	0.08	0.003 **
AGEs, A.U.	0.16	−5.6	5.9	0.96
Carotenoids, O.D.	0.00	−0.003	0.003	0.99

*p*-values were estimated by using a mixed-effects regression model. * and ** indicate significance levels at 5% (*p* < 0.05) and 1% (*p* < 0.01), respectively. IOP, intraocular pressure; IOPg, Goldmann-correlated IOP; ORA, ocular response analyzer; CI, confidence interval; M/F, male/female; BMI, body mass index; sBP, systolic blood pressure; dBP, diastolic blood pressure; PR, pulse rate; bpm, beats per minute; AGEs, advanced glycation end products; A.U., arbitrary unit; O.D., optical density.

**Table 6:** Multivariate analysis of possible associations between ORA-measured IOPcc and demographic parameters.

Parameters	Estimate	Lower 95% CI	Upper 95% CI	*p*-Value
Age, years	0.00	−0.03	0.03	0.95
Sex, M/F	0.40	−0.01	0.81	0.053
BMI, kg/m^2^	0.13	0.02	0.24	0.03 *
sBP, mmHg	0.03	0.01	0.06	0.015 *
dBP, mmHg	0.02	−0.02	0.05	0.41
PR, bpm	0.04	0.01	0.07	0.011 *
AGEs, A.U.	0.13	−5.5	5.7	0.96
Carotenoids, O.D.	0.00	−0.002	0.00	0.67

*p*-values were estimated by using a mixed-effects regression model. * indicates significance level at 5% (*p* < 0.05). IOP, intraocular pressure; IOPcc, corneal compensated IOP; ORA, ocular response analyzer; CI, confidence interval; M/F, male/female; BMI, body mass index; sBP, systolic blood pressure; dBP, diastolic blood pressure; PR, pulse rate; bpm, beats per minute; AGEs, advanced glycation end products; A.U., arbitrary unit; O.D., optical density.

**Table 7:** Multivariate analysis of possible associations between ORA-measured CH and demographic parameters.

Parameters	Estimate	Lower 95% CI	Upper 95% CI	*p*-Value
Age, years	−0.03	−0.04	−0.03	<0.0001 **
Sex, M/F	−0.22	−0.34	−0.11	<0.0001 **
BMI, kg/m^2^	0.00	−0.03	0.03	0.92
sBP, mmHg	0.00	−0.01	0.01	0.78
dBP, mmHg	−0.01	−0.02	0.00	0.28
PR, bpm	0.00	−0.01	0.01	0.86
AGEs, A.U.	0.03	−1.5	1.6	0.97
Carotenoids, O.D.	0.00	−0.001	0.00	0.14

*p*-values were estimated by using a mixed-effects regression model. ** indicates significance level at 1% (*p* < 0.01). CH, corneal hysteresis; ORA, ocular response analyzer; CI, confidence interval; M/F, male/female; BMI, body mass index; sBP, systolic blood pressure; dBP, diastolic blood pressure; PR, pulse rate; bpm, beats per minute; AGEs, advanced glycation end products; A.U., arbitrary unit; O.D., optical density.

**Table 8:** Multivariate analysis of possible associations between ORA-measured CRF and demographic parameters.

Parameters	Estimate	Lower 95% CI	Upper 95% CI	*p*-Value
Age, years	−0.04	−0.05	−0.03	<0.0001 **
Sex, M/F	−0.14	−0.27	0.00	0.04 *
BMI, kg/m^2^	0.04	0.01	0.08	0.02 *
sBP, mmHg	0.01	0.00	0.02	0.003 **
dBP, mmHg	0.00	−0.01	0.01	0.76
PR, bpm	0.02	0.01	0.03	0.003 **
AGEs, A.U.	0.18	−1.7	2.0	0.85
Carotenoids, O.D.	0.00	−0.001	0.00	0.32

*p*-values were estimated by using a mixed-effects regression model. * and ** indicate significance levels at 5% (*p* < 0.05) and 1% (*p* < 0.01), respectively. CRF, corneal resistance factor; ORA, ocular response analyzer; CI, confidence interval; M/F, male/female; BMI, body mass index; sBP, systolic blood pressure; dBP, diastolic blood pressure; PR, pulse rate; bpm, beats per minute; AGEs, advanced glycation end products; A.U., arbitrary unit; O.D., optical density.

**Table 9:** Comparison of demographic parameters and ORA parameters among groups stratified by quartiles of AGE scores.

Parameters	Q1	Q2	Q3	Q4	*p*-Value
Range	Low–0.377	0.377–0.423	0.423–0.47	0.47–High	
Age, years					
Mean ± SD	67.7 ± 0.7	68.5 ± 0.7	67.7 ± 0.7	71.3 ± 0.7	0.002 **
Range	18–88	21–91	19–96	18–96	
Sex					
Male, n (%)	144 (44)	167 (53)	205 (60)	188 (65)	<0.0001 **
Female, n (%)	186 (56)	148 (47)	138 (40)	102 (35)	
BMI, kg/m^2^					
Mean ± SD	22.3 ± 0.2	23.0 ± 0.2	23.1 ± 0.2	23.8 ± 0.2	<0.0001 **
Range	15.8–31.9	15.2–41.1	12.1–38.8	16.9–37.6	
sBP, mmHg					
Mean ± SD	146.1 ± 1.1	144.9 ± 1.2	144.5 ± 1.1	147.2 ± 1.3	0.45
Range	88–201	97–209	81–250	107–217	
dBP, mmHg					
Mean ± SD	82.0 ± 0.8	81.8 ± 0.8	80.7 ± 0.8	82.6 ± 0.8	0.41
Range	47–116	47–122	46–142	45–134	
PR, bpm					
Mean ± SD	75.1 ± 0.7	74.2 ± 0.7	75.8 ± 0.7	74.6 ± 0.7	0.37
Range	38–115	50–108	48–128	45–116	
Carotenoids, O.D.					
Mean ± SD	367.2 ± 7.0	366.2 ± 7.1	331.7 ± 6.9	283.9 ± 7.4	<0.0001 **
Range	106–782	70.7–748	101.5–708.5	103–668.5	
IOPg, mmHg					
Mean ± SD	15.0 ± 0.3	15.1 ± 0.4	15.7 ± 0.3	15.2 ± 0.4	0.47
Range	2.8–45.1	3.0–51.7	2.1–37.3	2.3–51.0	
IOPcc, mmHg					
Mean ± SD	16.7 ± 0.3	17.0 ± 0.4	17.7 ± 0.3	17.1 ± 0.4	0.22
Range	9.1–48.1	8.4–52.3	4.3–43.0	4.2–51.9	
CH, mmHg					
Mean ± SD	9.3 ± 0.1	9.2 ± 0.1	9.0 ± 0.1	9.1 ± 0.1	0.057
Range	3.1–12.8	3.2–14.3	3.3–13.6	2.1–14.0	
CRF, mmHg					
Mean ± SD	9.4 ± 0.1	9.3 ± 0.1	9.3 ± 0.1	9.2 ± 0.1	0.81
Range	3.5–15.2	4.1–17.2	4.1–14.5	4.9–16.7	

Comparison of characteristics stratified by the quartiles of the AGEs using one-way analysis of variance for continuous data and using the Cochran–Armitage trend test for categorical data. ** indicates significance level at 1% (*p* < 0.01). ORA, ocular response analyzer; AGEs, advanced glycation end products; SD, standard deviation; BMI, body mass index; sBP, systolic blood pressure; dBP, diastolic blood pressure; PR, pulse rate; bpm, beats per minute; O.D., optical density; IOP, intraocular pressure measured by Goldmann applanation tonometry; IOPcc, corneal compensated IOP; IOPg, Goldmann-correlated IOP; CH, corneal hysteresis; CRF, corneal resistance factor.

**Table 10:** Comparison of demographic subject parameters and ORA parameters among groups stratified by quartiles of carotenoid score.

Parameters	Q1	Q2	Q3	Q4	*p*-Value
Range	Low–249	249–318.3	318.3–413	413–High	
Age, years					
Mean ± SD	66.6 ± 0.7	68.4 ± 0.7	69.0 ± 0.7	70.9 ± 0.7	0.0002 *
Range	18–96	19–90	18–91	40–96	
Sex					
Male, *n* (%)	232 (70)	181 (58)	140 (44)	149 (47)	<0.0001 **
Female, *n* (%)	98 (30)	131 (42)	176 (56)	169 (53)	
BMI, kg/m^2^					
Mean ± SD	23.7 ± 0.2	23.5 ± 0.2	22.5 ± 0.2	22.3 ± 0.2	<0.0001 **
Range	12.1–41.1	15.3–38.8	16.7–35.0	15.8–32.9	
sBP, mmHg					
Mean ± SD	143.9 ± 1.2	148.1 ± 1.2	144.8 ± 1.2	145.3 ± 1.2	0.13
Range	81–217	98–250	95–243	94–224	
dBP, mmHg					
Mean ± SD	82.8 ± 0.8	83.5 ± 0.8	80.2 ± 0.7	80.6 ± 0.8	0.017 *
Range	45–142	47–135	46–129	53–122	
PR, cpm					
Mean ± SD	76.1 ± 0.7	74.2 ± 0.7	74.4 ± 0.7	75.0 ± 0.7	0.32
Range	45–116	38–112	52–128	50–126	
AGEs, A.U.					
Mean ± SD	0.44 ± 0.004	0.43 ± 0.004	0.41 ± 0.004	0.40 ± 0.004	<0.0001 **
Range	0.20–1.08	0.25–0.68	0.23–0.58	0.20–0.56	
IOPg, mmHg					
Mean ± SD	15.6 ± 0.3	15.3 ± 0.4	15.0 ± 0.4	14.2 ± 0.4	0.86
Range	2.1–51.7	2.3–46.8	3.9–45.1	3.0–48.7	
IOPcc, mmHg					
Mean ± SD	17.3 ± 0.3	17.0 ± 0.4	16.9 ± 0.4	17.2 ± 0.3	0.93
Range	4.3–52.3	4.2–51.9	8.1–48.1	8.4–50.8	
CH, mmHg					
Mean ± SD	9.3 ± 0.1	9.3 ± 0.1	9.1 ± 0.1	9.0 ± 0.1	0.22
Range	2.5–14.0	2.1–14.0	3.1–13.6	3.2–14.3	
CRF, mmHg					
Mean ± SD	9.5 ± 0.1	9.4 ± 0.1	9.2 ± 0.1	9.1 ± 0.1	0.18
Range	5.0–17.2	3.5–14.3	4.1–15.2	4.0–15.5	

Comparison of characteristics stratified by the quartiles of the skin carotenoids using one-way analysis of variance for continuous data and using the Cochran–Armitage trend test for categorical data. * and ** indicate significance levels at 5% (*p* < 0.05) and 1% (*p* < 0.01), respectively. ORA, ocular response analyzer; SD, standard deviation; BMI, body mass index; sBP, systolic blood pressure; dBP, diastolic blood pressure; PR, pulse rate; bpm, beats per minute; AGEs, advanced glycation end products; A.U., arbitrary unit; IOP, intraocular pressure measured by Goldmann applanation tonometry; IOPcc, corneal compensated IOP; IOPg, Goldmann-correlated IOP; CH, corneal hysteresis; CRF, corneal resistance factor.

## Data Availability

The raw data supporting the conclusions of this article will be made available by the authors on request.

## Abbreviations

The following abbreviations are used in this manuscript:

AGEs	Advanced Glycation End Products
A.U.	Arbitrary Unit
BMI	Body Mass Index
bpm	Beats Per Minute
CH	Corneal Hysteresis
CI	Confidence Interval
CRF	Corneal Resistance Factor
dBP	Diastolic Blood Pressure
IOP	Intraocular Pressure
IOPcc	Corneal Compensated Intraocular Pressure
IOPg	Goldmann-Correlated Intraocular Pressure
O.D.	Optical Density
ORA	Ocular Response Analyzer
PR	Pulse Rate
sAF	Skin Autofluorescence
sBP	Systolic Blood Pressure
SD	Standard Deviation
